# Characterization of protein-interaction networks in tumors

**DOI:** 10.1186/1471-2105-8-224

**Published:** 2007-06-27

**Authors:** Alexander Platzer, Paul Perco, Arno Lukas, Bernd Mayer

**Affiliations:** 1 Institute for Theoretical Chemistry, University of Vienna, Waehringer Strasse 17, A-1090 Vienna, Austria; 2 emergentec biodevelopment GmbH, Rathausstrasse 5/3, A-1010 Vienna, Austria

## Abstract

**Background:**

Analyzing differential-gene-expression data in the context of protein-interaction networks (PINs) yields information on the functional cellular status. PINs can be formally represented as graphs, and approximating PINs as undirected graphs allows the network properties to be characterized using well-established graph measures.

This paper outlines features of PINs derived from 29 studies on differential gene expression in cancer. For each study the number of differentially regulated genes was determined and used as a basis for PIN construction utilizing the Online Predicted Human Interaction Database.

**Results:**

Graph measures calculated for the largest subgraph of a PIN for a given differential-gene-expression data set comprised properties reflecting the size, distribution, biological relevance, density, modularity, and cycles. The values of a distinct set of graph measures, namely *Closeness Centrality*, *Graph Diameter*, *Index of Aggregation*, *Assortative Mixing Coefficient*, *Connectivity*, *Sum of the Wiener Number*, *modified Vertex Distance Number*, and *Eigenvalues *differed clearly between PINs derived on the basis of differential gene expression data sets characterizing malignant tissue and PINs derived on the basis of randomly selected protein lists.

**Conclusion:**

Cancer PINs representing differentially regulated genes are larger than those of randomly selected protein lists, indicating functional dependencies among protein lists that can be identified on the basis of transcriptomics experiments. However, the prevalence of hub proteins was not increased in the presence of cancer. Interpretation of such graphs in the context of robustness may yield novel therapies based on synthetic lethality that are more effective than focusing on single-action drugs for cancer treatment.

## Background

The "omics" revolution has dramatically increased the amount of data available for characterizing intracellular events at the cellular level. The main experimental methodologies responsible for this development have included differential gene expression analysis for recording mRNA concentration profiles, and proteomics for providing data on protein abundance [1,2]. Each technique generates data related to a defined intracellular aspect, such as differential-gene-expression profiles at the transcriptional level, and currently the main focus is on interlinking the various data sources generated by high-throughput screening and array technologies. The concept of systems biology is grounded on such heterogeneous data sources, and also includes the use of homolog information from other systems [3]. Methodologies following the framework of systems biology have increasingly been used to study complex diseases. For example, Hornberg and colleagues discussed the importance of the network topology of protein interactions to selecting drug targets for improving cancer therapy [4].

We have recently outlined a computational analysis workflow aimed at characterizing cellular events at a functional level, which includes the use of differential gene expression and proteomics data, analysis of transcriptional control, and coregulation via joint transcription factor modules, further complemented by protein interaction and functional pathway data [5]. A major goal of such analysis workflows is to decipher biological functioning at the level of protein interactions [6,7]; that is, to elucidate concerted processes by integrating diverse data sources that by themselves do not provide a functional context.

There are several experimental techniques for directly addressing protein-protein interactions, with the yeast two-hybrid system being the most commonly used [8]. The yeast two-hybrid approach can be used to identify protein interactions in vivo, with other techniques such as surface plasmon resonance being performed in a nonbiological environment, but still being useful for providing binding constants [9]. Other technologies involve protein arrays for parallel screening of protein interactions [10]. A recent review has discussed the different methodological approaches [11].

Public-domain databases have been established for making protein-protein-interaction data readily accessible. The Online Predicted Human Interaction Database (OPHID) is a collection of human protein-protein interactions assembled from other databases and complemented by homolog interactions identified in other organisms [12]. The OPHID database used in the present study (as at February 2006) included 41,785 interactions covering 8487 unique proteins of the human proteome. Unfortunately, the database contains only about 20% of the human proteome (presently representing about 39,000 sequences with a unique GI number). Generally, a literature bias is inherent in such interaction data due to disease associated genes and proteins being subject to more detailed analysis, also with respect to protein interactions.

Information on pairwise protein interactions as provided by the OPHID can be used to delineate protein interaction networks (PINs), which are usually represented as undirected graphs. Routines have been published for automatically generating and visualizing such interaction graphs [13,14], where the nearest-neighbor expansion as proposed by Chen and colleagues [15] is a useful approximation for extended graph construction when dealing with the sparse data sets typical of biological systems. Such routines can be used to directly extract PINs utilizing a list of proteins assembled on the basis of differentially expressed genes. If the functional context at the level of protein interactions is represented by the differential gene expression data, this should also be reflected by the characteristics of resulting PINs. Characteristics in this context include both quantitative measures (e.g., the number of nodes found for the largest subgraph) as well as qualitative measures in the biological context (e.g., the identification of hub proteins).

Like many real-world networks, biological networks are scale-free in nature, with the majority of nodes showing a low degree of connectivity, complemented by some highly connected nodes serving as hubs [16,17]. The connectivity, size, and topology of individual PINs are massively influenced by the number of hub proteins involved [18]. However, Lu and colleagues found in a murine asthma model that gene expression of the hub proteins tend to be less affected by disease [19]. The next-most-important factor to determining the overall PIN topology are the simple building blocks – such as a three-node "feedforward loop" motif or a four-node "bi-fan" motif – that have been detected more frequently in transcriptional gene regulatory networks than in networks generated from randomly selected genes [20]. PINs have been recently reviewed by Barabasi and Oltvai [21].

Various groups have applied network analysis to gene data sets associated with cancer. Jonsson and Bates reported very recently that proteins associated with cancer show an increased number of interacting partners in the interactome, reflecting their increased centrality in the PIN [22]. Wachi et al. specifically investigated the role of the interactome of genes differentially regulated in lung cancer [23]. That group found increased connectivity for these genes, in agreement with the findings of Jonsson and Bates. Tuck and colleagues analyzed transcriptional regulatory networks consisting of transcription factors and their target proteins [24]. Genes differentially regulated between acute myeloid leukemia and acute lymphoblastic leukemia were significantly closer in the network as compared to randomly generated gene lists. The analogous result was observed for genes differentially regulated in breast cancer patients. On a more general level, Xu and Li showed that disease-associated genes as listed in the OMIM database [25] tend to interact with other disease-associated genes [26].

The present paper provides a systematic analysis of properties computed for PINs represented as graphs, as exemplified by an extensive set of differential gene expression profiles covering various tumors. The primary hypothesis was that differential gene expression analysis provides systematic data on concerted events in malignant tissue [27], and these systematic data should also be present at the level of protein interactions, in contrast to network properties computed on the basis of randomly generated protein lists.

The formal representation of PINs as undirected graphs makes it possible to utilize a variety of well-established graph measures. Junker and colleagues recently presented a tool for exploring centralities in biological networks, named CentiBiN [28]. CentiBiN can calculate various graph measures, including closeness, betweenness, and eccentricity in protein networks. Jonsson and Bates demonstrated that proteins mutated in cancer showed an increased number of interactions [22]. Another study analyzed protein communities in PINs that were reported as being involved in metastatic processes [29]. Also, Jeong and colleagues were able to identify hub proteins in the PIN that are centrally linked to cell survival [30].

We have computed 22 individual graph measures for 29 tumor-associated differential gene expression data sets that reflect the following graph properties: size, distribution, relevance, density, modularity, and cycles. These graph measures provide a detailed characterization of the differential gene-expression data represented at the level of protein interactions.

## Results

A mean of 90 genes (SD = 74 genes, range = 13–300 genes) were identified as significantly differentially regulated for each transcriptomics experiment, and these genes were selected for constructing the entire graph for each given data set. Table 1 lists the number of differentially regulated genes (*N*), the number of nodes in graph (*G*), as well as the number of nodes in the largest subgraph (*G'*) for the 29 studies. Furthermore, the characteristics of the individual studies as included in the Oncomine database [31] are listed, including study author, tumor type, class comparison, and number of samples analyzed.

**Table 1:** Gene-expression studies and graph measures

**Study no.**	**Study author**	**cancer type**	**class I**	**class II**	**No. of Samples**	**N**	**G**	**G'**	**Size (3)**	**distribution (2)**	**relevance (3)**	**density (8)**	**modularity (3)**	**circles (3)**	**total (22)**
1	Rosenwald et al.	Leukemia	Blood B cell, Blood T cells, Cell Line, Cord Blood B cells, Cord Blood T cells, Diffuse Large Cell, Follicular Lymphoma, Nonblastic Cell Line, Thymic T cells, Tonsil GC B	Chronic Lymphocytic Leukemia	118	264	426	384	3	2	3	6	3	1	18
2	Segal et al.	Soft Tissue Cancer	Cell Line	Tumor	81	156	252	209	3	2	1	6	3	2	17
3	Rosenwald et al.	Diffuse Large B- Cell Lymphoma – Dlbcl Subgroup	Activated B-Cell-like DLBCL, Type III B-Cell-like DLBCL	Germinal-Center B- Cell-like	240	115	189	165	3	2	2	6	1	2	16
4	Rosenwald et al.	Diffuse Large B- Cell Lymphoma – Dlbcl Subgroup	Activated B-Cell-like DLBCL, Germinal-Center B-Cell-like	Type III B-Cell-like DLBCL	240	129	208	182	3	2	1	6	2	2	16
5	Welsh et al.	Ovary – Type	Normal Ovary	Ovarian Adenocarcinoma	32	96	153	128	3	2	1	6	1	1	14
6	Beer et al.	Lung – Type	Non-neoplastic Lung	Lung Adenocarcinoma	96	158	267	247	3	1	0	6	3	1	14
7	Notterman et al.	Colon – Type	Normal Colon	Ovarian Adenocarcinoma	36	41	62	44	3	1	1	5	1	2	13
8	Higgins et al.	Kidney – Type	Normal Kidney	Clear Renal Cell Carcinoma	29	62	96	76	3	1	2	5	1	1	13
9	Khan et al.	Small Round Blue Cell Tumor/Cell Line	Cell Line	Tumor Sample	86	126	196	155	3	0	1	5	2	1	12
10	Lancaster et al.	Ovary – Type	Ovary	Ovarian Adenocarcinoma	34	106	169	135	3	1	1	5	1	1	12
11	Welsh et al.	Prostate – Type	Normal Prostate	Prostate Cancer	34	50	77	58	3	1	0	4	1	2	11
12	Singh et al.	Prostate – Type	Prostate	Prostate Carcinoma	102	300	469	409	2	1	1	3	2	2	11
13	Liang et al.	Brain – Type	Normal Brain	Glioblastoma Multiforme	33	53	86	70	3	1	0	5	1	1	11
14	Higgins et al.	Kidney – Type	Angiomyolipoma, Chromophobe Renal Cell Carcinoma, Granular Renal Cell Carcinoma, Oncocytoma, Papillary Renal Cell Carcinoma	Normal Kidney	44	55	87	64	3	1	0	4	1	1	10
15	Sperger et al.	Germ Cell – Type	Normal Testis	Seminoma	37	219	342	279	3	1	0	4	1	1	10
16	Shai et al.	Brain – Type	Normal White Matter	Glioblastoma Multiforme	32	56	84	63	3	1	0	4	1	1	10
17	Rickman et al.	Brain – Type	Normal Neocortex of Temporal Lobe	Glioma	51	46	67	42	3	0	0	3	1	1	8
18	Rosenwald et al.	Lymphoid – Type	Normal Blood CD19+ B-Cells, Normal Germinal Center B-Cells	Diffuse Large B-Cell Lymphoma	284	37	60	32	2	0	0	4	1	0	7
19	Frierson et al.	Salivary Gland – Type	Normal Salivary Gland	Adenoid Cystic Carcinoma of Salivary Gland	22	70	104	72	1	1	0	2	1	1	6
20	Bhattacharjee et al.	Lung – Type	Normal Lung	Lung Adenocarcinoma	156	128	195	149	2	0	0	1	1	1	5
21	Bhattacharjee et al.	Lung – Type	Normal Lung	Squamous Cell Lung Carcinoma	38	111	167	123	0	1	0	0	1	1	3
22	Lenburg et al.	Kidney – Type	Normal Kidney	Renal Clear Cell Carcinoma	18	13	14	3	0	0	0	1	0	0	1
23	Garber et al.	Lung – Type	Normal Lung	Squamous Cell Carcinoma	19	26	34	5	0	0	0	0	1	0	1
24	Alon et al.	Colon – Type	Colon	Colon Adenocarcinoma	62	13	16	3	0	0	0	0	0	0	0
25	LaTulippe et al.	Prostate – Type	Non-neoplastic Prostate	Prostate Carcinoma	26	24	29	9	0	0	0	0	0	0	0
26	Iacobuzio- Donahue et al.	Pancreas – Type	Normal pancreas	Pancreatic Adenocarcinoma	17	80	106	35	0	0	0	0	0	0	0
27	Mutter et al.	Uterus – Type	Normal Endometrium	Endometrioid Adenocarcinoma	14	16	18	5	0	0	0	0	0	0	0
28	Bhattacharjee et al.	Lung – Type	Normal Lung	Small Cell Lung Cancer	23	17	20	7	0	0	0	0	0	0	0
29	Garber et al.	Lung – Type	Normal Lung	Lung Adenocarcinoma	46	45	58	9	0	0	0	0	0	0	0

Study number, study author, cancer type, class comparison, and number of samples for data from the Oncomine database. The number of differentially regulated genes (*N*), the number of nodes in graph *G*, the number of nodes in largest subgraph *G'*, and the number of measures per category outside the 2.5% lower and upper confidence limits as derived on the basis of randomly generated gene lists, and the total number of graph measures per study that fell outside the defined significance limits are also listed.

The mean number of nodes in *G *(after performing the nearest-neighbor expansion) was 140 (SD = 120 nodes, range = 14–469 nodes) for the 29 studies, with a mean of 109 nodes for the largest subgraph *G' *(SD = 110 nodes, range = 3–409 nodes). For seven of the studies there were less than 30 nodes in the largest subgraph. Measures related to size, distribution, biological relevance, density, modularity, and cycles were computed for each subgraph *G'*.

### Size measures

**Figure 1:**
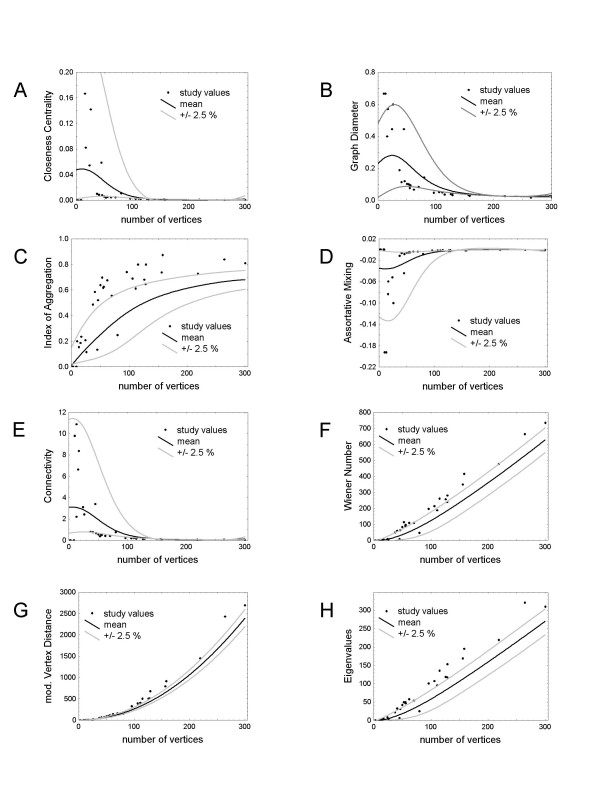
**Graph measures**. Graph measures (black dots) computed for the given differential gene expression data sets from 29 individual studies with between 10 and 300 genes. The following graph measures are presented: *Closeness Centrality *(**A**), *Graph Diameter *(**B**), *Index of Aggregation *(**C**), *Assortative Mixing Coefficient *(**D**), *Connectivity *(**E**), *Sum of the Wiener Number *(**F**), *modified Vertex Distance Number *(**G**) and *Eigenvalues *(**H**). The mean value (black curve) and the 2.5% lower and upper confidence limits (fitted graphs) based on randomly generated data sets are given for each graph measure.

We used three measures to characterize the graph size as reflected by the number of vertices, the graph expansion, and the length of the shortest path. All three measures – *Closeness Centrality*, *Graph Diameter*, and *Index of Aggregation *– were different for networks generated from gene lists derived from Oncomine than for randomly generated protein lists (Figure 1A,B and 1C), with networks derived on the basis of Oncomine data sets tending to be larger than networks derived on the basis of randomly generated protein sets.

### Distribution measures

We used two distribution measures in our analysis: the *Assortative Mixing Coefficient *and the *entropy of the distribution of edges*. The *Assortative Mixing Coefficient *uses the edge-to-edge distribution, whereas the *entropy of the distribution of edges *uses an entropic term reflecting the distinct number of edges per node. We found that the *Assortative Mixing Coefficient *was significantly higher in Oncomine networks than in random networks (Figure 1D).

### Biological-relevance measures

Three of the 22 computed measures focused on vertices in the network that were biologically relevant. All of the measures took the shortest path between two vertices in a given network into account. Highly connected proteins, frequently called hub proteins, usually show high *Betweenness*. Joy et al. demonstrated the importance of vertices with high *Betweenness *but low connectivity in the yeast PIN [32]. Interestingly, none of the three computed biological-relevance measures differed significantly between Oncomine networks and randomly generated networks.

### Density measures

Eight of the 22 measures utilized in this study addressed aspects of graph density, including *Connectivity*, *Graph Centrality*, *Community*, and *Sum of the Wiener Number*. The numbers of edges and vertices, lengths of shortest paths, and walks on edges were key elements in calculating these measures. Two of the eight measures (*Connectivity *and the *Sum of the Wiener Number*) differed between Oncomine and random data sets (Figure 1E and 1F), and these are influenced by the size of the graph. Oncomine networks are generally larger but less dense than randomly generated networks.

### Modularity measures

We calculated three measures reflecting modularity, mainly associated with the number of edges, dilation, and shortest path lengths. One of the computed measures, namely the *modified Vertex Distance Number*, differed between Oncomine networks and randomly generated networks (Figure 1G). This measure is highly correlated to *Closeness Centrality*, which is also based on the sum of shortest paths between two vertices.

### Cycles measures

The three measures implemented related to graph cycles were the *Cyclic Coefficient*, *Subgraph Centrality*, and *Eigenvalues*. The *Eigenvalues*, calculated from the adjacency matrix of the graph, differed between randomly generated data sets and Oncomine (Figure 1H). *Eigenvalues*, like *Subgraph Centrality*, mainly depend on all cycles of the graph, but the two methods differ in the scaling of cycle sizes. The *Cyclic Coefficient *mainly depends on local short cycles.

To study the data sets at the level of the graph-measure categories, the 22 graph properties of each data set were checked for measures that significantly deviated from those of random graphs. Results of this evaluation are listed in Table 1, where the individual studies are sorted by the total number of graph measures that deviated significantly from those derived from random gene selections. The study that deviated the most from random selections related to leukemia, in which 18 of the 22 graph measures were different. On the other hand, in six studies none of the graph measures differed significantly from random selections. Tests of the correlation between the number of graph measures deviating from their respective values for random selections and the total number of genes differentially regulated (*r*^2 ^= 0.34, *p *< 0.05), the total number of nodes in graph *G *(*r*^2 ^= 0.38, *p *< 0.05), and the total number of nodes in the largest subgraph *G' *(*r*^2 ^= 0.43, *p *< 0.05) revealed the dependence on number of nodes selected and the degree of deviation from random selections. This correlation was significantly affected by the small graphs analyzed, since studies resulting in subgraph sizes of less than 10 do not provide conclusive graph measures.

Interestingly, the number of samples analyzed for differential gene expression was not significantly correlated with the number of statistically significant differentially regulated genes found (*r*^2 ^= 0.09, *p *= 0.12), nor with the number of graph measures deviating from the randomly generated reference sets (*r*^2 ^= 0.11, *p *> 0.05).

## Discussion

We characterized PINs derived from 29 gene-expression profiles of various tumors (as listed in Table 1) by computing 22 graph measures (as listed in Table 2). In general, the values of the graph measures did not depend on the type of microarray used in the analysis (cDNA arrays or Affymetrix Gene Chips). The small number of individual data sets per cancer type made it impossible to delineate a correlation between graph measures and tissue type. Interestingly, the number of samples used was not correlated with the number of statistically significant differentially expressed genes, and also not with the number of graph measures deviating from random selections. Under the assumption of comparable sample processing, expression results are strongly affected by the tissue and cancer type, and to a lesser extent on the number of samples per group.

**Table 2:** Formal representation of graph measures

**Name**	**Class**	**Definition**	**Description**	**Ref.**
***Closeness Centrality***	size	CCi=1∑jd(i,j) MathType@MTEF@5@5@+=feaafiart1ev1aaatCvAUfKttLearuWrP9MDH5MBPbIqV92AaeXatLxBI9gBaebbnrfifHhDYfgasaacH8akY=wiFfYdH8Gipec8Eeeu0xXdbba9frFj0=OqFfea0dXdd9vqai=hGuQ8kuc9pgc9s8qqaq=dirpe0xb9q8qiLsFr0=vr0=vr0dc8meaabaqaciaacaGaaeqabaqabeGadaaakeaacqWGdbWqcqWGdbWqdaWgaaWcbaGaemyAaKgabeaakiabg2da9maalaaabaGaeGymaedabaWaaabuaeaacqWGKbazcqGGOaakcqWGPbqAcqGGSaalcqWGQbGAcqGGPaqkaSqaaiabdQgaQbqab0GaeyyeIuoaaaaaaa@3C7C@	*d*(*i,j*) is the length of the shortest path between vertices *i *and *j*. The sum of *CC*_*i *_over all vertices gives the total *Closeness Centrality *of a given subgraph.	**[42]**
***Graph Diameter***	size	GD=max⁡(d(i,j))N MathType@MTEF@5@5@+=feaafiart1ev1aaatCvAUfKttLearuWrP9MDH5MBPbIqV92AaeXatLxBI9gBaebbnrfifHhDYfgasaacH8akY=wiFfYdH8Gipec8Eeeu0xXdbba9frFj0=OqFfea0dXdd9vqai=hGuQ8kuc9pgc9s8qqaq=dirpe0xb9q8qiLsFr0=vr0=vr0dc8meaabaqaciaacaGaaeqabaqabeGadaaakeaacqWGhbWrcqWGebarcqGH9aqpdaWcaaqaaiGbc2gaTjabcggaHjabcIha4jabcIcaOiabdsgaKjabcIcaOiabdMgaPjabcYcaSiabdQgaQjabcMcaPiabcMcaPaqaaiabd6eaobaaaaa@3D82@	*d*(*i,j*) is the length of the shortest path between vertices *i *and *j*. *GD *is computed for all pairs (*i,j*), and reflects the longest path identified.	**[43]**
***Index of Aggregation***	size	IoA=AB MathType@MTEF@5@5@+=feaafiart1ev1aaatCvAUfKttLearuWrP9MDH5MBPbIqV92AaeXatLxBI9gBaebbnrfifHhDYfgasaacH8akY=wiFfYdH8Gipec8Eeeu0xXdbba9frFj0=OqFfea0dXdd9vqai=hGuQ8kuc9pgc9s8qqaq=dirpe0xb9q8qiLsFr0=vr0=vr0dc8meaabaqaciaacaGaaeqabaqabeGadaaakeaacqWGjbqscqWGVbWBcqWGbbqqcqGH9aqpdaWcaaqaaiabdgeabbqaaiabdkeacbaaaaa@3367@	*A *is the total number of vertices in the subgraph, and *B *is the total number of all given vertices in the graph.	**[15]**
***Assortative Mixing Coefficient***	distribution		*k*_*1 *_and *k*_*2 *_are the counts of edges of two vertices connected by a given edge. This measure reflects the edge-to-edge distribution over all edges of a graph.	**[44]**
***Entropy of the distribution of edges***	distribution	H=−∑kp(k)ln⁡p(k) MathType@MTEF@5@5@+=feaafiart1ev1aaatCvAUfKttLearuWrP9MDH5MBPbIqV92AaeXatLxBI9gBaebbnrfifHhDYfgasaacH8akY=wiFfYdH8Gipec8Eeeu0xXdbba9frFj0=OqFfea0dXdd9vqai=hGuQ8kuc9pgc9s8qqaq=dirpe0xb9q8qiLsFr0=vr0=vr0dc8meaabaqaciaacaGaaeqabaqabeGadaaakeaacqWGibascqGH9aqpcqGHsisldaaeqbqaaiabdchaWjabcIcaOiabdUgaRjabcMcaPiGbcYgaSjabc6gaUjabdchaWjabcIcaOiabdUgaRjabcMcaPaWcbaGaem4AaSgabeqdcqGHris5aaaa@3EF4@	*k *is the count of edges of one vertex, and *p*(*k*) is the ratio of vertices that have *k *edges.	**[45]**
***Betweenness***	biological relevance	B=∑i∈V∑j,kσ(j,i,k)σ(j,k)N MathType@MTEF@5@5@+=feaafiart1ev1aaatCvAUfKttLearuWrP9MDH5MBPbIqV92AaeXatLxBI9gBaebbnrfifHhDYfgasaacH8akY=wiFfYdH8Gipec8Eeeu0xXdbba9frFj0=OqFfea0dXdd9vqai=hGuQ8kuc9pgc9s8qqaq=dirpe0xb9q8qiLsFr0=vr0=vr0dc8meaabaqaciaacaGaaeqabaqabeGadaaakeaacqWGcbGqcqGH9aqpdaWcaaqaamaaqafabaWaaabuaeaadaWcaaqaaGGaciab=n8aZjabcIcaOiabdQgaQjabcYcaSiabdMgaPjabcYcaSiabdUgaRjabcMcaPaqaaiab=n8aZjabcIcaOiabdQgaQjabcYcaSiabdUgaRjabcMcaPaaaaSqaaiabdQgaQjabcYcaSiabdUgaRbqab0GaeyyeIuoaaSqaaiabdMgaPjabgIGiolabdAfawbqab0GaeyyeIuoaaOqaaiabd6eaobaaaaa@4C63@	*σ*(*j,i,k*) is the total number of shortest connections between vertices *j *and *k*, where each shortest connection has to pass vertex *i*, and *σ*(*j,k*) is the total number of shortest connections between *j *and *k*. We computed *σ*(*j,i,k*) and *σ*(*j,k*) for the entire OPHID graph, but then only used vertices also present in the subgraph generated on the basis of a given gene-expression data set.	**[42]**
***Betweenness of all selected Vertices***	biological relevance		As for *Betweenness*, but considering all selected vertices.	**[42]**
***Stress Centrality***	biological Relevance	StC=∑i∈V∑j,kσ(j,i,k) MathType@MTEF@5@5@+=feaafiart1ev1aaatCvAUfKttLearuWrP9MDH5MBPbIqV92AaeXatLxBI9gBaebbnrfifHhDYfgasaacH8akY=wiFfYdH8Gipec8Eeeu0xXdbba9frFj0=OqFfea0dXdd9vqai=hGuQ8kuc9pgc9s8qqaq=dirpe0xb9q8qiLsFr0=vr0=vr0dc8meaabaqaciaacaGaaeqabaqabeGadaaakeaacqWGtbWucqWG0baDcqWGdbWqcqGH9aqpdaaeqbqaamaaqafabaacciGae83WdmNaeiikaGIaemOAaOMaeiilaWIaemyAaKMaeiilaWIaem4AaSMaeiykaKcaleaacqWGQbGAcqGGSaalcqWGRbWAaeqaniabggHiLdaaleaacqWGPbqAcqGHiiIZcqWGwbGvaeqaniabggHiLdaaaa@46AA@	*σ*(*j,i,k*) is the total number of shortest connections between vertices *j *and *k*, where each shortest connection has to pass vertex *i*.	**[42]**
***Connectivity***	density	C=AB MathType@MTEF@5@5@+=feaafiart1ev1aaatCvAUfKttLearuWrP9MDH5MBPbIqV92AaeXatLxBI9gBaebbnrfifHhDYfgasaacH8akY=wiFfYdH8Gipec8Eeeu0xXdbba9frFj0=OqFfea0dXdd9vqai=hGuQ8kuc9pgc9s8qqaq=dirpe0xb9q8qiLsFr0=vr0=vr0dc8meaabaqaciaacaGaaeqabaqabeGadaaakeaacqWGdbWqcqGH9aqpdaWcaaqaaiabdgeabbqaaiabdkeacbaaaaa@30E9@	*A *is the total number of edges realized in a given graph, and *B *is the maximum number of edges possible.	**[43]**
***Clustering Coefficient***	density	CLUSTi=AB MathType@MTEF@5@5@+=feaafiart1ev1aaatCvAUfKttLearuWrP9MDH5MBPbIqV92AaeXatLxBI9gBaebbnrfifHhDYfgasaacH8akY=wiFfYdH8Gipec8Eeeu0xXdbba9frFj0=OqFfea0dXdd9vqai=hGuQ8kuc9pgc9s8qqaq=dirpe0xb9q8qiLsFr0=vr0=vr0dc8meaabaqaciaacaGaaeqabaqabeGadaaakeaacqWGdbWqcqWGmbatcqWGvbqvcqWGtbWucqWGubavdaWgaaWcbaGaemyAaKgabeaakiabg2da9maalaaabaGaemyqaeeabaGaemOqaieaaaaa@372E@	*A *is the total number of edges between the nearest neighbors of vertex *i*, and *B *is the maximum number of possible edges between the nearest neighbors of vertex *i*. The sum of *CLUST*_*i *_over all vertices gives the total *Clustering Coefficient *of a given subgraph.	**[46]**
***Number of edges divided by the number of vertices***	density	NeNv=AB MathType@MTEF@5@5@+=feaafiart1ev1aaatCvAUfKttLearuWrP9MDH5MBPbIqV92AaeXatLxBI9gBaebbnrfifHhDYfgasaacH8akY=wiFfYdH8Gipec8Eeeu0xXdbba9frFj0=OqFfea0dXdd9vqai=hGuQ8kuc9pgc9s8qqaq=dirpe0xb9q8qiLsFr0=vr0=vr0dc8meaabaqaciaacaGaaeqabaqabeGadaaakeaacqWGobGtcqWGLbqzcqWGobGtcqWG2bGDcqGH9aqpdaWcaaqaaiabdgeabbqaaiabdkeacbaaaaa@34EC@	*A *is the total number of edges in a given graph, and *B *is the number of selected vertices in a given graph.	**-**
***Community***	density	Comm=AB MathType@MTEF@5@5@+=feaafiart1ev1aaatCvAUfKttLearuWrP9MDH5MBPbIqV92AaeXatLxBI9gBaebbnrfifHhDYfgasaacH8akY=wiFfYdH8Gipec8Eeeu0xXdbba9frFj0=OqFfea0dXdd9vqai=hGuQ8kuc9pgc9s8qqaq=dirpe0xb9q8qiLsFr0=vr0=vr0dc8meaabaqaciaacaGaaeqabaqabeGadaaakeaacqWGdbWqcqWGVbWBcqWGTbqBcqWGTbqBcqGH9aqpdaWcaaqaaiabdgeabbqaaiabdkeacbaaaaa@3516@	*A *is the total number of edges, where both connected vertices are in the given subgraph, and *B *is the total number of edges, where one connected vertex is in the subgraph and the other vertex is outside it.	**[47]**
***Entropy***	density	H(G)=∑v∈V,i(v)>=2(i(v)−1)∗log⁡(|E|−|V|+1i(v)−1) MathType@MTEF@5@5@+=feaafiart1ev1aaatCvAUfKttLearuWrP9MDH5MBPbIqV92AaeXatLxBI9gBaebbnrfifHhDYfgasaacH8akY=wiFfYdH8Gipec8Eeeu0xXdbba9frFj0=OqFfea0dXdd9vqai=hGuQ8kuc9pgc9s8qqaq=dirpe0xb9q8qiLsFr0=vr0=vr0dc8meaabaqaciaacaGaaeqabaqabeGadaaakeaacqWGibascqGGOaakcqWGhbWrcqGGPaqkcqGH9aqpdaaeqaqaaiabcIcaOiabdMgaPjabcIcaOiabdAha2jabcMcaPiabgkHiTiabigdaXiabcMcaPiabgEHiQiGbcYgaSjabc+gaVjabcEgaNjabcIcaOmaalaaabaGaeiiFaWNaemyrauKaeiiFaWNaeyOeI0IaeiiFaWNaemOvayLaeiiFaWNaey4kaSIaeGymaedabaGaemyAaKMaeiikaGIaemODayNaeiykaKIaeyOeI0IaeGymaedaaaWcbaGaemODayNaeyicI4SaemOvayLaeiilaWIaemyAaKMaeiikaGIaemODayNaeiykaKIaeyOpa4Jaeyypa0JaeGOmaidabeqdcqGHris5aOGaeiykaKcaaa@6057@	where |*E*| is the total number of edges, |*V*| is the total number of vertices, and *i*(*v*) is the number of edges of vertex *v*.	**[48]**
***Graph Centrality***	density	GCi=1max⁡(d(i,j)) MathType@MTEF@5@5@+=feaafiart1ev1aaatCvAUfKttLearuWrP9MDH5MBPbIqV92AaeXatLxBI9gBaebbnrfifHhDYfgasaacH8akY=wiFfYdH8Gipec8Eeeu0xXdbba9frFj0=OqFfea0dXdd9vqai=hGuQ8kuc9pgc9s8qqaq=dirpe0xb9q8qiLsFr0=vr0=vr0dc8meaabaqaciaacaGaaeqabaqabeGadaaakeaacqWGhbWrcqWGdbWqdaWgaaWcbaGaemyAaKgabeaakiabg2da9maalaaabaGaeGymaedabaGagiyBa0MaeiyyaeMaeiiEaGNaeiikaGIaemizaqMaeiikaGIaemyAaKMaeiilaWIaemOAaOMaeiykaKIaeiykaKcaaaaa@3EDC@	max(*d*(*i,j*)) is the length of the shortest path between vertices *i *and *j *for a given vertex *i*.	**[42]**
***Number of walks of length n***	density	NW=∑NWi MathType@MTEF@5@5@+=feaafiart1ev1aaatCvAUfKttLearuWrP9MDH5MBPbIqV92AaeXatLxBI9gBaebbnrfifHhDYfgasaacH8akY=wiFfYdH8Gipec8Eeeu0xXdbba9frFj0=OqFfea0dXdd9vqai=hGuQ8kuc9pgc9s8qqaq=dirpe0xb9q8qiLsFr0=vr0=vr0dc8meaabaqaciaacaGaaeqabaqabeGadaaakeaacqWGobGtcqWGxbWvcqGH9aqpdaaeabqaaiabd6eaojabdEfaxnaaBaaaleaacqWGPbqAaeqaaaqabeqaniabggHiLdaaaa@35FA@	*NW*_*i *_is one walk with a length of *n *edges in the subgraph.	**[43]**
***Sum of the Wiener Number***	density	Wi=12∗∑i,jd(i,j) MathType@MTEF@5@5@+=feaafiart1ev1aaatCvAUfKttLearuWrP9MDH5MBPbIqV92AaeXatLxBI9gBaebbnrfifHhDYfgasaacH8akY=wiFfYdH8Gipec8Eeeu0xXdbba9frFj0=OqFfea0dXdd9vqai=hGuQ8kuc9pgc9s8qqaq=dirpe0xb9q8qiLsFr0=vr0=vr0dc8meaabaqaciaacaGaaeqabaqabeGadaaakeaacqWGxbWvdaWgaaWcbaGaemyAaKgabeaakiabg2da9maalaaabaGaeGymaedabaGaeGOmaidaaiabgEHiQmaaqafabaGaemizaqMaeiikaGIaemyAaKMaeiilaWIaemOAaOMaeiykaKcaleaacqWGPbqAcqGGSaalcqWGQbGAaeqaniabggHiLdaaaa@3FB1@	*d*(*i,j*) is the length of the shortest path between vertices *i *and *j*. We computed the *Sum of the Wiener Number *for each vertex.	**[43]**
***Total number of triangles of a subgraph and its dilation***	Modularity		Given a subgraph *g *of graph *G*, the complement of *g*, denoted as *g*, is the subgraph implied by the set of vertices *N*(*g*) = *N*(*G*)\*N*(*g*) The dilation of *g *is the subgraph *δ*(*g*) implied by the vertices in *g *plus the vertices directly connected to a vertex in *g*. The coat of nearest neighbors of the subgraph is defined as *DN*(*g*) = *δ*(*g*)\*N*(*g*) The set of all valid triangles for *g *is defined as *VT*(*g*) = {*x*,*y*,*z *| (*x*,*y*,*z *∈ *N*(*δ*(*g*)) ^ (*x*,*y*),(*y*,*z*),(*z*,*x*) ∈ *E*(*δ*(*g*))) ∩ (*x *∈ *N*(*g*) ^ *z *∈ *DN*(*g*))} where *N *is the number of vertices and *E *is the number of edges in the graph. The result for a subgraph *g *is the total number of elements in *VT*(g).	**[42]**
***Localized Modularity***	modularity	LM=|Einside||Ewithin the (direct) neighbors|∗|Einside|∗|Eto the outside||Ewithin the (direct) neighbors|2 MathType@MTEF@5@5@+=feaafiart1ev1aaatCvAUfKttLearuWrP9MDH5MBPbIqV92AaeXatLxBI9gBaebbnrfifHhDYfgasaacH8akY=wiFfYdH8Gipec8Eeeu0xXdbba9frFj0=OqFfea0dXdd9vqai=hGuQ8kuc9pgc9s8qqaq=dirpe0xb9q8qiLsFr0=vr0=vr0dc8meaabaqaciaacaGaaeqabaqabeGadaaakeaacqWGmbatcqWGnbqtcqGH9aqpdaWcaaqaaiabcYha8jabbweafnaaBaaaleaacqqGPbqAcqqGUbGBcqqGZbWCcqqGPbqAcqqGKbazcqqGLbqzaeqaaOGaeiiFaWhabaGaeiiFaWNaeeyrau0aaSbaaSqaaiabbEha3jabbMgaPjabbsha0jabbIgaOjabbMgaPjabb6gaUjabbccaGiabbsha0jabbIgaOjabbwgaLjabbccaGiabcIcaOiabbsgaKjabbMgaPjabbkhaYjabbwgaLjabbogaJjabbsha0jabcMcaPiabbccaGiabb6gaUjabbwgaLjabbMgaPjabbEgaNjabbIgaOjabbkgaIjabb+gaVjabbkhaYjabbohaZbqabaGccqGG8baFaaGaey4fIOYaaSaaaeaacqGG8baFcqqGfbqrdaWgaaWcbaGaeeyAaKMaeeOBa4Maee4CamNaeeyAaKMaeeizaqMaeeyzaugabeaakiabcYha8jabgEHiQiabcYha8jabbweafnaaBaaaleaacqqG0baDcqqGVbWBcqqGGaaicqqG0baDcqqGObaAcqqGLbqzcqqGGaaicqqGVbWBcqqG1bqDcqqG0baDcqqGZbWCcqqGPbqAcqqGKbazcqqGLbqzaeqaaOGaeiiFaWhabaGaeiiFaWNaeeyrau0aaSbaaSqaaiabbEha3jabbMgaPjabbsha0jabbIgaOjabbMgaPjabb6gaUjabbccaGiabbsha0jabbIgaOjabbwgaLjabbccaGiabcIcaOiabbsgaKjabbMgaPjabbkhaYjabbwgaLjabbogaJjabbsha0jabcMcaPiabbccaGiabb6gaUjabbwgaLjabbMgaPjabbEgaNjabbIgaOjabbkgaIjabb+gaVjabbkhaYjabbohaZbqabaGccqGG8baFdaahaaWcbeqaaiabikdaYaaaaaaaaa@B47C@	where |*E*| is the total number of edges.	**[49]**
***modified Vertex Distance Number***	modularity	mVD=∑i,j∈V,i≠jV1d(i,j)2 MathType@MTEF@5@5@+=feaafiart1ev1aaatCvAUfKttLearuWrP9MDH5MBPbIqV92AaeXatLxBI9gBaebbnrfifHhDYfgasaacH8akY=wiFfYdH8Gipec8Eeeu0xXdbba9frFj0=OqFfea0dXdd9vqai=hGuQ8kuc9pgc9s8qqaq=dirpe0xb9q8qiLsFr0=vr0=vr0dc8meaabaqaciaacaGaaeqabaqabeGadaaakeaacqWGTbqBcqWGwbGvcqWGebarcqGH9aqpdaaeWbqaamaalaaabaGaeGymaedabaGaemizaqMaeiikaGIaemyAaKMaeiilaWIaemOAaOMaeiykaKYaaWbaaSqabeaacqaIYaGmaaaaaaqaaiabdMgaPjabcYcaSiabdQgaQjabgIGiolabdAfawjabcYcaSiabdMgaPjabgcMi5kabdQgaQbqaaiabdAfawbqdcqGHris5aaaa@4931@	*d*(*i,j*) is the length of the shortest path between vertices *i *and *j*. For this measure, *i *and *j *are all selected from V.	**-**
***Eigenvalues***	cycles	EV=∑j|ERj|2 MathType@MTEF@5@5@+=feaafiart1ev1aaatCvAUfKttLearuWrP9MDH5MBPbIqV92AaeXatLxBI9gBaebbnrfifHhDYfgasaacH8akY=wiFfYdH8Gipec8Eeeu0xXdbba9frFj0=OqFfea0dXdd9vqai=hGuQ8kuc9pgc9s8qqaq=dirpe0xb9q8qiLsFr0=vr0=vr0dc8meaabaqaciaacaGaaeqabaqabeGadaaakeaacqWGfbqrcqWGwbGvcqGH9aqpdaaeqbqaaiabcYha8jabdweafjabdkfasnaaBaaaleaacqWGQbGAaeqaaOGaeiiFaW3aaWbaaSqabeaacqaIYaGmaaaabaGaemOAaOgabeqdcqGHris5aaaa@3B61@	*ER*_*j *_is the real part of the *j*-th *Eigenvalue *for the adjacency matrix of the given subgraph.	**[50]**
***Subgraph Centrality***	cycles	SC=1N∑i=1N∑k=1∞(Ak)iik! MathType@MTEF@5@5@+=feaafiart1ev1aaatCvAUfKttLearuWrP9MDH5MBPbIqV92AaeXatLxBI9gBaebbnrfifHhDYfgasaacH8akY=wiFfYdH8Gipec8Eeeu0xXdbba9frFj0=OqFfea0dXdd9vqai=hGuQ8kuc9pgc9s8qqaq=dirpe0xb9q8qiLsFr0=vr0=vr0dc8meaabaqaciaacaGaaeqabaqabeGadaaakeaacqWGtbWucqWGdbWqcqGH9aqpdaWcaaqaaiabigdaXaqaaiabd6eaobaadaaeWbqaamaaqahabaWaaSaaaeaacqGGOaakcqWGbbqqdaahaaWcbeqaaiabdUgaRbaakiabcMcaPiabdMgaPjabdMgaPbqaaiabdUgaRjabcgcaHaaaaSqaaiabdUgaRjabg2da9iabigdaXaqaaiabg6HiLcqdcqGHris5aaWcbaGaemyAaKMaeyypa0JaeGymaedabaGaemOta4eaniabggHiLdaaaa@4917@	*A *is the adjacency matrix. We computed *SC *for *k *[1,99].	**[42]**
***Cyclic Coefficient***	cycles	θ(i)=2ki∗(ki−1)∗∑j,k1Si(j,k)θ=1/N∗θ(i) MathType@MTEF@5@5@+=feaafiart1ev1aaatCvAUfKttLearuWrP9MDH5MBPbIqV92AaeXatLxBI9gBaebbnrfifHhDYfgasaacH8akY=wiFfYdH8Gipec8Eeeu0xXdbba9frFj0=OqFfea0dXdd9vqai=hGuQ8kuc9pgc9s8qqaq=dirpe0xb9q8qiLsFr0=vr0=vr0dc8meaabaqaciaacaGaaeqabaqabeGadaaakeaafaqaaeGabaaabaacciGae8hUdeNaeiikaGIaemyAaKMaeiykaKIaeyypa0ZaaSaaaeaacqaIYaGmaeaacqWGRbWAdaWgaaWcbaGaemyAaKgabeaakiabgEHiQiabcIcaOiabdUgaRnaaBaaaleaacqWGPbqAaeqaaOGaeyOeI0IaeGymaeJaeiykaKcaaiabgEHiQmaaqafabaWaaSaaaeaacqaIXaqmaeaacqWGtbWudaWgaaWcbaGaemyAaKgabeaakiabcIcaOiabdQgaQjabcYcaSiabdUgaRjabcMcaPaaaaSqaaiabdQgaQjabcYcaSiabdUgaRbqab0GaeyyeIuoaaOqaaiab=H7aXjabg2da9iabigdaXiabc+caViabd6eaojabgEHiQiab=H7aXjabcIcaOiabdMgaPjabcMcaPaaaaaa@590D@	*S*_*i *_is the smallest possible cycle of vertex *i *and two of its neighboring vertices *k*. The total *Cyclic Coefficient *for all vertices *N *is then given as *θ*	**[42]**

Name, formal representation, and short description of graph measures computed for the categories of size, distribution, biological relevance, density, modularity, and cycles.

We assigned the graph measures to the following categories: size, distribution, biological relevance, density, modularity, and cycles. The individual graph measures that showed significant differences (defined as identifying at least 50% of gene-expression experiments outside the 2.5% lower and upper confidence limits computed on the basis of randomly generated data sets) between cancer networks and networks based on randomly generated data sets were *Closeness Centrality*, *Graph Diameter*, *Index of Aggregation*, *Assortative Mixing Coefficient*, *Connectivity*, *Sum of the Wiener Number*, *modified Vertex Distance Number*, and *Eigenvalues*.

All three measures associated with the size of the graph differed significantly between tumor networks and randomly generated networks. The *Index of Aggregation *was on average higher in tumor networks, indicating dependencies between proteins involved in cancer, as also proposed by Chen et al. in the context of Alzheimer disease [15]. This increased connectivity is also consistent with data obtained by Jonsson et al. [22]. However, it is likely that the bias in OPHID interactions toward disease-associated genes contributes to these findings. The values of both *Graph Diameter *and *Closeness Centrality *were significantly lower in tumor networks. This finding was also reported by Yu and colleagues for networks solely including highly expressed genes in the yeast interactome [33]. Low *Closeness Centrality *values for tumor networks may initially appear surprising, but relative large size of the largest subgraphs in tumor networks (on average close to 80% of all nodes of *G *are also part of *G'*) makes higher *Closeness Centrality *values harder to obtain. The largest subgraph of tumor networks also more elongated shortest paths between nodes.

One measure of the distribution category, the *Assortative Mixing Coefficient*, differed significantly in tumor networks. This coefficient is influenced by both the number of hub proteins and the number of edges, and a large number of hub proteins is correlated with an unequal distribution in the number of edges. The *Assortative Mixing Coefficient *is directly proportional to the number of edges and inversly proportional to the number of hub proteins. According to Jonsson and colleagues, tumor networks contain numerous hub proteins [22]. However, our data generally indicate the presence of a small number of edges per node, and no evidence for a large number of hub proteins.

The *Sum of the Wiener Number *characterizes the density of the graph. The significantly higher values of this measure in tumor networks indicate larger graphs, which is consistent with the observed *Index of Aggregation*. We found that the *Connectivity *was lower in the largest subgraphs of tumor networks. This may be also due to the largest subgraphs of tumor networks being on average larger than the subgraphs of randomly generated gene lists, corresponding to low values of *Closeness Centrality*.

The *modified Vertex Distance Number *is also influenced by the sum of shortest paths between two vertices, but in contrast to *Closeness Centrality*, all vertices in the OPHID network are considered. A higher *modified Vertex Distance Number *in tumor networks indicates higher connectivity and modularity in Oncomine networks. Finally, higher *Eigenvalues *values indicate the presence of fewer cycles in tumor networks.

Our analysis of 29 studies on differential gene expression in cancer has revealed a general tendency toward large subgraphs without the presence of explicit hubs. Comparing the graph measures between the individual gene expression studies and randomly selected genes provided a heterogeneous picture. Gene-expression studies resulting in a low number of statistically significant differentially regulated sequences (and consequently small subgraphs) do not support an interpretation at the level of PINs (see expression studies 22–29 in Table 1) as performed in this study: for small subgraphs the variance of graph measures determined for randomly selected gene lists is high, which prevents identification of significant differences of small subgraphs derived on the basis of differential gene-expression data.

## Conclusion

The usefulness of analyzing topological characteristics of cancer networks for supporting drug targeting was recently highlighted by Hornberg and colleagues [4]. We based our study on a diverse set of cancer types, and have identified characteristics of cancer networks from differential-gene-expression data. In particular, measures of graph size deviated significantly from those for graphs constructed from random gene selections. Genes showing significant differential expressions in cancer appear to be interlinked also at the level of PINs. However, we were not able to identify hub proteins from the given data, or nodes exhibiting high *Betweenness*. Such nodes have been considered as primary targets for therapeutic interventions.

Extended graphs with a low density may indicate a network with high robustness – in contrast to networks containing hub proteins. This points to a different approach for identifying therapeutic intervention, namely synthetic lethality. This concept originates in classical genetics, where only the combination of two specific mutations leads to cell death. In metabolic networks a single node deletion can often be bypassed by different routes in the pathway. Combining this with a second deletion in that alternative pathway may only then result in lethality [34]. Analysis of the given PINs with respect to functional pathways and their potential bypass routes has the potential to identify synhetically lethal protein target combinations, as has been shown experimentally in yeast [35].

## Methods

### Databases

We used the OPHID [12] to derive information on human protein-protein interactions. This database contains information on protein-interaction pairs, where each protein is given by its Swiss-Prot identifier. We mapped the Swiss-Prot identifiers on the corresponding Gene Symbols so as to link gene-expression data sets, which mapped 8487 Swiss-Prot entries to 6033 different Gene Symbols. Among the protein-interaction sources used by the OPHID, we included HPRD (Human Protein Reference Database) [36], MINT (Molecular Interaction Database) [37], RikenBIND and RikenDIP [38], BIND (Biomolecular Interaction Network Database, [39], and MIPS (Munich Information Center for Protein Sequences) [40]. These data sets are mostly based on experimental evidence, which is further supported by expert reviews based on the scientific literature. We did not include interactions from other sources of low-to-medium quality that are also listed and indicated as such in the OPHID.

The OPHID provides interaction information in the form of object A interacting with object B. This information can be used to derive interaction graphs when providing an identifier list (A, B, ..., N), as resulting from the analysis of differential-gene-expression data.

We used Oncomine as a central repository for differential-gene-expression data [31]. This database provides an extensive collection of gene expression data on cancer, and compares various types and subgroups. A total of 962 raw data sets were identified in Oncomine (as at April 2006). We manually selected all gene expression studies where the malignant tissue was compared to a reference (either healthy tissue or a cell line). We initially selected 40 individual experiments covering tumors of 17 different tissues (4 B-cell, 1 bladder, 2 colon, 2 endometrium, 2 ovary, 5 brain, 1 liver, 1 leukemia, 9 lung, 1 multicancer, 3 kidney, 1 pancreas, 4 prostate, 1 salivary gland, 1 testis, 1 thyroid, and 1 soft-tissue tumor), of which 17 used cDNA arrays and 23 used Affymetrix Gene Chips. The mean number of available features per study was 11459 (range = 1988–44928 features).

We extracted each file and processed the raw data according to the following scheme: The two groups per study were analyzed at the level of individual genes by computing a probability value for the differential expression of a particular gene in that given experiment. Multiple testing was accounted for by using the Holm-Sidak step-down test and setting the significance level to 0.05 [41]. This procedure yield a mean of 278 genes from each study (range = 2–1838 genes). From the initial 40 gene expression data sets, 29 showed between 10 and 300 differentially expressed genes (mean = 90 genes), and these studies were included in subsequent analyses.

Each of the 29 selected differential gene expression studies was represented by a list of genes exhibiting significant differential regulation when comparing expression values for the group of tumor samples and the group of reference samples. Each gene on these lists was represented by its Gene Symbol, allowing a direct match with the protein interaction data as derived from the OPHID.

### Graph construction

Protein interaction graphs (*G*) were constructed for each gene list of the 29 selected gene-expression studies based on OPHID interaction data utilizing the nearest-neighbor expansion. This procedure built edges between the nodes of entries A and B of a given gene list if the interaction between A and B was directly encoded in the OPHID, or if one element X was identified in the OPHID, allowing the construction of an interaction of the type A - X - B, where X was not listed in the gene expression data set [15].

For each gene list, entire graph *G *comprising *n *subgraphs *G' *was constructed on the basis of genes in the initial list and their nearest neighbors in the PIN. *G' *is defined as a graph whose vertices and edges form subsets of the vertices and edges of *G*.

Gene lists derived from analyzing differential gene expression might be linked on the level of coregulation and protein interactions. To quantitatively assess such dependencies, the graph properties of PINs derived on the basis of randomly selected gene lists were computed as follows: Proteins encoded by randomly selected gene lists exhibit a background level of protein interactions, and we analyzed graph measures characterizing gene expression data sets with respect to random data sets. One thousand random gene sets containing between 10 and 300 genes were picked in steps of 10. For each of these gene sets, the largest subgraph *G' *was generated again following the nearest-neighbor expansion as outlined above, and the graph measures were computed for each *G'*. This procedure yielded the mean value and 2.5% lower and upper confidence limits for each graph measure for each data set size represented by the 1000 individual data sets.

### Graph measures and data evaluation

The graph measures for each largest subgraph *G' *were then determined for each Oncomine data set as well as for random data sets. Table 2 lists all of the applied graph measures. (Software for computing these properties on the basis of given Gene Symbol lists is available from the authors upon request.) The graph measures derived for Oncomine data sets were then interpreted in the context of the measure scales based on random data sets. A graph measure was considered as interesting in the context of cancer associated networks if at least 50% of the 29 Oncomine experiments showed this measure to be outside the 2.5% lower and upper confidence limits as computed on the basis of the randomly generated data sets.

## Authors' contributions

BM and PP designed the study. AP extended the concept, developed the software, and performed all the calculations. AP, PP, AL, and BM contributed to data interpretation and writing the manuscript. All authors read and approved the final manuscript.
